# Survival Analysis in Single N2 Station Lung Adenocarcinoma: The Prognostic Role of Involved Lymph Nodes and Adjuvant Therapy

**DOI:** 10.3390/cancers13061326

**Published:** 2021-03-16

**Authors:** Marco Chiappetta, Filippo Lococo, Giovanni Leuzzi, Isabella Sperduti, Emilio Bria, Leonardo Petracca Ciavarella, Felice Mucilli, Pier Luigi Filosso, Giovannibattista Ratto, Lorenzo Spaggiari, Francesco Facciolo, Stefano Margaritora

**Affiliations:** 1 Università Cattolica del Sacro Cuore, 00168 Rome, Italy; Filippo.lococo@policlinicogemelli.it (F.L.); emilio.bria@policlinicogemelli.it (E.B.); leonardo.petracca@gmail.com (L.P.C.); stefano.margaritora@policlinicogemelli.it (S.M.); 2 Thoracic Surgery, Fondazione Policlinico Universitario A. Gemelli, IRCCS, 00168 Rome, Italy; 3 Thoracic Surgery, Unit Fondazione IRCCS Istituto Nazionale dei Tumori, 20133 Milan, Italy; giovanni.leuzzi@istitutotumori.mi.it; 4 Biostatistics, Regina Elena National Cancer Institute—IRCCS, 00100 Rome, Italy; isabella.sperduti@ifo.gov.it; 5 Medical Oncology, IRCCS Fondazione Policlinico Universitario A. Gemelli, 00168 Rome, Italy; 6 Department of General and Thoracic Surgery, University Hospital “SS. Annunziata”, 66100 Chieti, Italy; fmucilli@unich.it; 7 Department of Thoracic Surgery, University of Turin, San Giovanni Battista Hospital, 10126 Turin, Italy; pierluigi.filosso@unito.it; 8 Division of Thoracic Surgery, IRCCS AOU “San Martino” IST, 16132 Genoa, Italy; giovannibattista.ratto@gmail.com; 9 Thoracic Surgery Division, European Institute of Oncology, University of Milan, 20141 Milan, Italy; lorenzo.spaggiari@ieo.it; 10 Thoracic Surgery, Regina Elena National Cancer Institute, 00100 Rome, Italy; francesco.facciolo@ifo.gov.it

**Keywords:** NSCLC, adenocarcinoma, lymph node, surgery, adjuvant therapy

## Abstract

**Simple Summary:**

Lung adenocarcinoma is the most common histology in non-small cell lung cancer that has a large variety of histological and also pathological presentations. However, mediastinal lymph node involvement remains one of the most important prognosticators in these patients, and treatment can vary from upfront surgery to induction therapy or definitive radio-chemotherapy. One of the most intriguing issues regards the identification of the most appropriate treatment for a patient and one of the parameters that may indicate upfront surgical resection is the presence of single mediastinal station involvement. Moreover, another interesting argument regards the adjuvant therapy indication for these patients. For these reasons, we investigated the survival outcome of patients who underwent surgical resection for lung adenocarcinoma and single mediastinal station involvement, with the aim to investigate the prognostic factors in this class of patients.

**Abstract:**

Background: Prognostic factors in patients with single mediastinal station (sN2) involvement continues to be a debated issue. Methods: Data on 213 adenocarcinoma patients with sN2 involvement and who had undergone complete anatomical lung resection and lymphadenectomy, were retrospectively reviewed. Clinical and pathological characteristics together with adjuvant therapy (AD) and node (N) status classifications (number of resected nodes (#RN), number of metastatic nodes (#MN), and node ratio (#MN/#RN = NR) were analyzed. Results: Univariable analysis confirmed that age (0.009), #MN (0.009), NR (0.003), #N1 involved stations (*p* = 0.003), and skip metastases (*p* = 0.005) were related to overall survival (OS). Multivariable analysis confirmed, as independent prognostic factors, age <66 years and NR with a three-year OS (3YOS) of 78.7% in NR < 10% vs. 46.6% in NR > 10%. In skip metastases, NR (HR 2.734, 95% CI 1.417–5.277, *p* = 0.003) and pT stage (HR2.136, 95% CI 1.001–4.557, *p* = 0.050) were confirmed as independent prognostic factors. AD did not influence the OS of patients with singular positive lymph nodes (*p* = 0.41), while in patients with multiple lymph nodes and AD, a significantly better 3YOS was demonstrated, i.e., 49.1% vs. 30% (*p* = 0.004). In patients with N2 + N1 involvement, age (*p* = 0.002) and AD (*p* = 0.022) were favorable prognostic factors. Conclusions: Adenocarcinoma patients with single N2 station involvement had a favorable outcome in the case of skip metastases and low NR. Adjuvant therapy improves survival with multiple nodal involvement, while its role in single node involvement should be clarified.

## 1. Introduction

The optimal therapeutic approach in patients with mediastinal lymph node involvement in non-small cell lung cancer is a very debated issue, with different possible strategies varying from upfront surgery to definitive radio- and chemotherapy to induction therapy plus surgery with different schedules [1,2].

For this reason, multidisciplinary assessment and identification of favorable prognostic factors is fundamental to the identification of the best therapeutic approach. For a better stratification of the node (N) parameter, the 8th edition of the tumor node metastases staging system (TNM) has proposed a sub-classification substantially based on the number of involved stations [3], with the definition of two categories of non-small cell lung cancer (NSCLC) patients, i.e., single N2 station involvement with (N2a2) or without N1 involvement (N2a1, skip metastases).

Indeed, one of the clinical factors that could be an indicator for surgery is the presence of single N2 station involvement, i.e., a patient group presenting a quite good prognosis as compared with multiple N2 stations [1,2,4]. However, the proposal presents some limitations related to the overlapping of the survival curves, especially when comparing patients with skip metastases or single N2 station involvement with hilar metastases.

Moreover, considering other studies, the prognosis of patients with skip metastases remains controversial, and despite the presence of a slight survival advantage as compared with other types of N2 involvement, there is still no definitive conclusion on this issue [4,5].

Starting from this evidence, different studies have analyzed the validity of the proposal, and some studies have confirmed overlapping curves [6,7], and possible confounding factors such as histology or the number of involved lymph nodes [7,8].

Indeed, in skip metastases adenocarcinoma, the prognosis seems to be better as compared with multiple hilar stations or single N2 station + hilar involvement, while in squamous cell carcinoma, the survival rate is poor in skip metastases as compared with multiple hilar stations involvement [7]. Similarly, other authors [8] have reported the prognostic role of the number of metastatic lymph nodes or better outcomes in the case of skip metastases, with significant differences among the same category (N1 or N2) or in single versus multiple lymph node involvement.

In consideration of these results, histology and the type of nodal involvement seem to be important factors in patients with single N2 station involvement, and their categorization may be useful for a better prognosis stratification.

The aims of this study are to analyze the survival outcome, the prognostic role of lymph node involvement type, and the prognostic role of the adjuvant therapy in patients affected by lung adenocarcinoma with single N2 station involvement, who have undergone surgical treatment.

## 2. Materials and Methods

**Figure 1:**
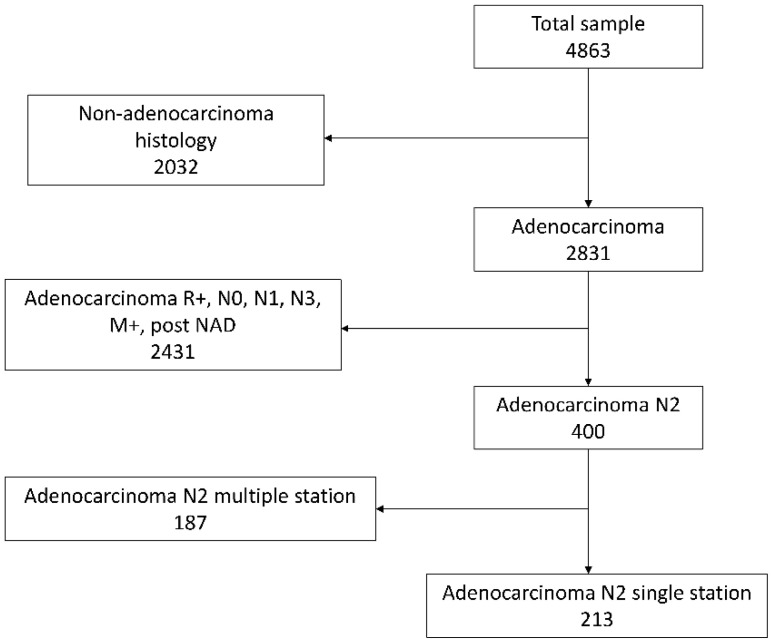
A flowchart showing patient selection for the study.

Data on 4863 patients affected by NSCLC, who underwent surgical treatment from January 2002 to December 2014 among 7 institutions were collected and retrospectively analyzed. Patients without N2 involvement, N3 or distant metastases, incomplete resections, or those who had undergone neoadjuvant therapy as well as patients with non-adenocarcinoma histology were excluded from the analysis (Figure 1).

Finally, the analysis was conducted on 213 adenocarcinoma patients with pathological single-station N2 involvement. According to the 8th TNM proposal, patients without N1 involvement were categorized as N2a1, while patients with single N2 + N1 involvement were categorized as N2a2. Although different centers were involved in this multicenter collaboration, the same policies in terms of preoperative workout were applied, including a whole-body computed tomography with contrast, 18-fluorodeoxyglucose positron emission tomography—CT (18F-FDG PET-CT) (when adopted and available in the involved center), and brain magnetic resonance in the case of suspected brain metastases. Lymph nodes larger than 1 cm on computed tomography or showing increased FDG uptake independently of the size were defined as metastatic nodes. As necessary, if pre-operative radiological evaluation was not exhaustive, in the case of N1 involvement or central tumors >3 cm, endobronchial ultrasound-guided transbronchial needle aspiration cytology or mediastinoscopy were performed for clinical staging.

Surgery was performed by certified thoracic surgeons and consisted of major anatomical lung resection (lobectomy, bilobectomy, or pneumonectomy) associated with lymphadenectomy (sampling or radical mediastinal lymph node dissection) according to the IASLC lymph node map and ESTS guidelines [9,10]. In particular, all patients underwent one of the following lymphadenectomies:

Sampling: The removal of one or more lymph nodes guided by preoperative or intraoperative findings which are thought to be representative,

Radical nodal dissection: All the mediastinal tissue containing the lymph nodes is dissected and removed systematically within anatomical landmarks.

Lymphadenectomy was limited in the case of hemodynamic instability or in the case of sticky lymph nodes non dissociable from the mediastinal structures as infective disease results (e.g., calcified nodes in tuberculosis patients).

In the case of lymph node fragmentation, lymph node parts were added to the corresponding node station for the histological analysis and counted as single node fragments. Pathological reports were reviewed and classified according to the 8th TNM edition [11].

Oncologists and radiotherapists indicated and administered adjuvant therapy (AD) in accordance with pathological stage, patients’ clinical conditions and clinical guidelines were validated during the study period [12]. In general, AD chemotherapy consisted of platinum-based therapy in association with a second chemotherapy drug depending on histology and patients’ clinical conditions, usually administered 4–8 weeks after surgery in 4 cycles every 28 days. Adjuvant radiotherapy was administered only in a few cases such as uncertain resection for the presence of extracapsular nodal involvement or resection margins close to the tumor [12]. There were no patients who received molecular targeted therapy or immune checkpoint inhibitors as AD.

Although some inter-institutional variation could have been present, follow-up was comprised of clinical visits, blood tests, and radiological examinations (computed tomography and positron emission tomography when available and indicated) every 3 to 6 months after surgery for the first 2 years postoperatively, and then every 6 months for 5 years.

Clinical and pathological characteristics (age, sex, pathological stage, kind of intervention, and grading) were analyzed together with adjuvant therapy and different N status classifications (number of resected nodes (#RN), number of metastatic nodes [#MN, node ratio (#MN/#RN), number of resected stations, number of positive hilar stations, and presence of skip metastases).

### Statistical Analysis

Descriptive analyses, including clinical and demographic characteristics of the patients, were analyzed through the median and the range for continuous variables and the absolute value and relative frequencies for categorical variables.

Survival curves were calculated by the Kaplan–Meier product-limit method from the date of surgery until relapse or death. The log-rank test was used to assess differences between subgroups. Significance was defined at the *p* ≤ 0.05 level. The hazard ratio (HR) and the 95% confidence interval (95% CI) were estimated using the Cox univariate model. A multivariate Cox proportional hazard model was developed using stepwise regression (forward selection) to compare the prognostic power of different factors. The enter limit and removal limit were *p* = 0.10 and *p* = 0.15, respectively. The assessment of interactions between significant investigation variables was taken into account when developing the multivariate model. Statistical evaluations were performed using SPSS (v. 21.0, SPSS Inc., Chicago, IL, USA)

## 3. Results

### 3.1. Overall

The final analysis was conducted on 213 patients, who met the inclusion criteria. Clinical and pathological characteristics are reported in Table 1.

Median follow-up was 33 (range 1–152) months. Median three-year disease-free survival (3Y-DFS) and OS (3YOS) results were 35 (CI 95% 27–42) and 46 (CI 95% 36–56) months, respectively. Skip metastases were present in 89 patients and adjuvant therapy was performed in the majority of patients (149, 69.9%).

Univariate analysis results showed that young age (0.009), low number of positive lymph nodes (0.009), low lymph node ratio (0.003), low number of N1 involved stations (*p* = 0.003), and presence of skip metastases (*p* = 0.005) were favorable prognostic factors for OS (Table 2).

**Figure 2:**
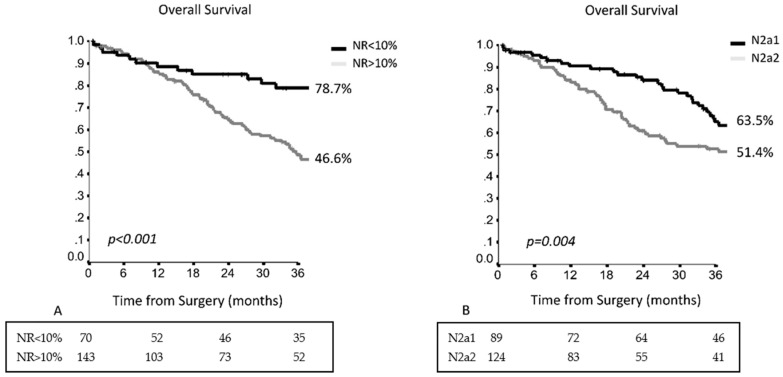
(**A**) The 3-year overall survival (OS) in the entire population in accordance with the node ratio (NR); (**B**) The 3-year OS in the entire population in accordance with the presence of skip metastases (N2a1) or concomitant hilar involvement (N2a2).

Multivariable analysis confirmed age <66 years (HR 1.808, 95% CI 1.220–2.662, *p* = 0.003) and NR (HR 2.761, 95% CI 1.683–4.530, *p* < 0.001) as independent favorable prognostic factors. In particular, an optimal cut-off for NR was identified, and in patients with node ratio <10%, the 3YOS result was 78.7% vs. 44.6% (*p* = 0.001) (Figure 2A and Table 2). In skip metastases, the 3YOS was 63.5% vs. 51.4% in N2a2 patients (Figure 2B).

**Figure 3:**
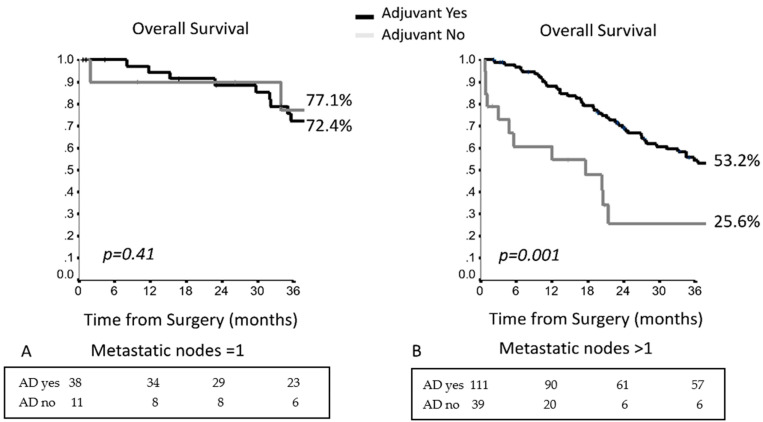
The 3-year OS in the entire population in accordance with administration of adjuvant therapy considering the number of metastatic lymph nodes; (**A**) Only one metastastic node; (**B**) Multiple lymph node involvement.

Considering the number of involved lymph nodes, in patients with single lymph node involvement, the administration of AD did not improve survival (Figure 3A), while in patients with multiple lymph node involvement who underwent AD, the 3YOS was significantly better as compared with those without AD, i.e., 53.2% vs. 25.6% (Figure 3B) (*p* = 0.001).

Regarding DFS, only tumor grading (*p* = 0.024) was a significant prognostic factor, while AD improved DFS in patients with multiple lymph node involvement (*p* = 0.02).

### 3.2. N2a1 Patients (Skip Metastases)

**Figure 4:**
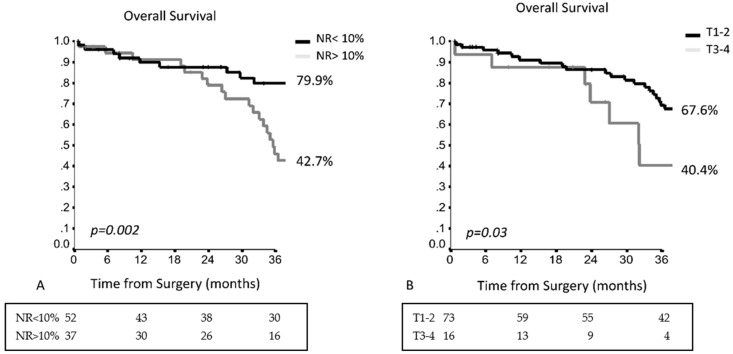
(**A**) The 3-year OS in patients with skip metastases in accordance with the lymph node ratio (NR); (**B**) The 3-year OS in patients with skip metastases in accordance with the pathological T-stage.

Dividing the patients based on concomitant N1 involvement, in patients with skip metastases, NR was an independent prognostic factor, with a 3YOS of 79.9% for patients with NR < 10%, while for patients with NR > 10% the 3YOS was 42.7% (HR 2.734, 95% CI 1.417–5.277, *p* = 0.003) (Figure 4A). Pathological T-stage was an independent prognostic factor with a 3YOS of 67.6% in T1–T2 vs. 40.4% in T3–T4 (HR 2.136, 95% CI 1.001–4.557, *p* = 0.050 (Figure 4B and Table S1).

Regarding adjuvant therapy, in the case of singular lymph node involvement, the difference in OS was not significant (*p* = 0.41), while, patients, in the case of multiple lymph node involvement who underwent AD, had a significantly better 3YOS, i.e., 49.1% vs. 30% (*p* = 0.004).

In these patients, the administration of adjuvant therapies resulted in a significant improvement in terms of DFS (HR 2.514, 95% CI 1.226–5.158, *p* = 0.012).

### 3.3. N2a2 Patients (Concomitant Hilar Involvement)

In patients with concomitant hilar involvement, a significant difference in terms of long-term survival was observed when stratifying by age (*p* = 0.002) and the administration of adjuvant therapy, with a 3YOS of 54.4% vs. 26.7% in patients with or without adjuvant therapy (*p* = 0.022, Table S2). Multivariable analysis confirmed the age (HR 2.013, 95% CI 1.194–3.939, *p* = 0.009) as the only independent prognostic factor. Considering the DFS, in the group of N2a2, the administration of adjuvant therapy result was an independent prognostic factor, with a 3YDFS of 53.4% vs. 24.2% (HR 2.219, 95% CI 1.051–4.685, *p* = 0.037).

## 4. Discussion

Our study shows that the type of lymph node involvement and administration of adjuvant therapy are important prognostic factors in patients with single mediastinal station involvement, a class of patients that is usually considered to have a favorable prognosis among all patients affected by non-small cell lung cancer with mediastinal involvement.

Summarizing in brief, our results show that the absence of N1 involvement, a low number of involved nodes, and a low NR were significantly correlated with a better survival rate, in addition, multivariate analysis confirmed only NR as an independent prognostic factor. Conversely, the number of resected lymph nodes was not a prognostic factor in this class of patients. This may be due to the patient characteristic of this cohort, with a high number of resected nodes (median of 17), which may be a sign of an appropriate lymphadenectomy. Moreover, previous studies have reported a prognostic value especially in early stages and with a relatively small number of resected nodes, which may be the signal of a limited and partial lymph node assessment, suggesting that a high number of harvested nodes may improve staging accuracy [7,13,14].

In line with other studies, we reported the effectiveness of the NR in patients with lymph node involvement [15,16] and specifically in patients with unexpected N2 involvement [17], and the effectiveness of this parameter in prognosis prediction was also confirmed in the specific subcategory with single-station involvement.

The efficacy of the NR was confirmed in the subgroup of patients with skip metastases, permitting a further stratification for these patients; in this subset of patients, a significant survival benefit may be achieved by the administration of adjuvant therapy.

The prognostic role of the number of metastatic nodes remains to be an intriguing issue, and when the entire population was considered, we observed a correlation with survival. Conversely, in accordance with the results of Katsumata et al. [8], the presence of single versus multiple positive lymph nodes did not show a statistical significance in patients with skip metastases. It is possible that this category presents an intrinsic favorable outcome due to the skip of hilar stations and relatively less nodal involvement.

Indeed, based on the anatomical lymphatic drainage, direct mediastinal involvement seems to be related to a direct connection to mediastinal nodes, without interception of hilar nodes. Although a codified route is present for the lymphatic drainage, crossing the hilar, and then the mediastinal lymph nodes, a direct connection to the mediastinal nodes is not common, occurring in only about 25% of anatomic cases [16]. Such direct drainage is more common in the upper lobes (about 35% of cases) as compared with the lower lobes (about 16%) and may be due to direct lymphatic vessels by-passing the hilar station or due to the lymphatics in the visceral pleura [18,19,20].

Therefore, it may be correct to talk about first lymph node involvement rather than skip metastases [18,19] as routinely performed in other malignancies (i.e., sentinel lymph node in breast cancer). This phenomenon could also explain overlapping curves considering the survival difference between patients with skip N2 metastases as compared with patients with multiple hilar station involvement [3,7]. Indeed, considering nodal involvement due to first intercepted lymph node, it is possible that the topographic classification results are inadequate for survival stratification, which explains the better survival outcomes reported for patients with skip metastases [4,5].

This concept was also reported in an analysis by Park et al. [21], which did not demonstrate differences in survival comparing N2a1 to different N1 patterns. We are in agreement with the authors’ considerations regarding the limitations of the anatomical classification and the need to consider other factors such as the number of lymph nodes or node ratio, especially if their use in preoperative work-up may be challenging.

In this study, we reported a 3YOS in skip metastases with singular nodal involvement of 77.1% when adjuvant therapy was administered and of 72.4% without AD involvement, similar to patients with N1 involvement reported in literature [3,7]. This result may be considered to be another indirect proof-of-concept of first intercepted lymph nodes, explaining the good prognosis in these patients despite mediastinal involvement, and, in our study, independently by T stage.

The prognostic role of the number of involved lymph nodes continues to be an intriguing issue with only recent studies that have analyzed its potential role showing promising results [8,16,21]. In particular, Katsumata et al. reported a significant survival stratification considering single versus multiple N1 node involvement, while in the case of N2 patients, the differences were not statistically significant [8]. Conversely, other studies have reported the effectiveness of categorizing patients based on the number of metastatic nodes, but associating N1 and N2 patients [14,21]. Starting from these experiences, it may be possible to better define patients with N2 involvement who may benefit from upfront surgery, indicating this approach for patients with single mediastinal metastatic lymph node.

However, one of the most important limitations of considering metastatic node numbers is the preoperative assessment difficulty. For this reason, parameters such as the NR may be considered especially for the pathological evaluation, trying to combine these factors with the optimal specific anatomical landmark.

In this study, we confirmed its validity in patients with single-station involvement, showing a survival stratification comparable to the proposed classification based on the concomitant hilar involvement.

Another interesting point regards the possibility of stratifying patients for adjuvant treatments. Indeed, adjuvant therapy is a fundamental treatment in patients with N2 involvement [1,2,17], with a proven survival benefit when administered.

However, few data are present regarding its potential benefits according to the number of involved stations or number of lymph nodes, which may better define an indication for adjuvant therapy. Legras et al. [18] reported worse survival in the case of adjuvant therapy administration, but this result may be due to the indication of AD in advanced stages (multiple N2 stations involvement) as compared with patients without N1 metastases.

We reported a survival benefit in terms of overall survival in the overall population, and also considering N2a1 and N2a2 subgroups, also confirming the importance of this approach for patients in the favorable group (skip metastases). In particular, in skip metastases, we found that AD was a predictor for DFS but not for OS, and interestingly, we noticed that it might influence the prognosis based on the type of nodal involvement.

Indeed, in these patients, in the case of singular lymph node involvement, AD did not ensure any survival advantage, while considering patients with multiple positive nodes, AD ensured a survival benefit of about the 25% at three years.

Different studies have reported the survival advantages of patients with skip metastases or single-station involvement [4,5,7,22,23], but only Yazgan et al. reported a survival advantage after adjuvant therapy [5]. However, in their study, the authors analyzed the outcome in a heterogeneous population of patients with N1 and/or N2 involvement, also including patients, who had undergone neoadjuvant therapy. In our study, we focused our attention on specific histology and the type of nodal involvement, confirming the importance of adjuvant therapy especially in two categories of patients with single-station involvement. Indeed, we found a survival improvement in the case of concomitant hilar involvement and in N2a1 patients with multiple nodes metastases, while in N2a1 patients with single metastatic node, AD did not influence survival.

These results also seem to be confirmed in our study considering the number of positive lymph nodes, suggesting that this parameter may also be useful for indicating adjuvant therapy to reduce the risk of treatment administration without clear survival benefit. To our knowledge, this is the first study reporting this kind of analysis; further studies with larger populations are needed to confirm our findings. However, our results are based on platinum-based chemotherapy, while it would be interesting to evaluate the role of tyrosin kinase inhibitors or immunotherapy in the AD setting, performing a tailored therapy in this class of patients.

Finally, age was an independent prognostic factor for survival in these patients, which is also in agreement with other studies that have analyzed this patient group [5,24], confirming that this is an important factor to consider before upfront surgery in the case of clinical N2 disease.

However, patient selection is a fundamental part of the surgical approach in the case of mediastinal involvement, and the inclusion of these parameters (age as well as performance status or Karnosky index) during multidisciplinary meeting [2,12,25] may represent another step for appropriate and tailored treatment strategies for these patients.

This study presents some (unavoidable) limitations that are common in similar studies on this topic. Firstly, due to its retrospective nature, it is difficult to obtain information regarding clinical course, including patients, who underwent upfront surgery for limited N2 disease, adjuvant therapy, or patients with unexpected N2 involvement. However, surgery indication and AD administration were in accordance with the study period and were performed based on different TNM classifications (6th and 7th) that presented differences, especially regarding the T3 and the T4 categories. However, in this study, we considered patients without pleural effusion that were recategorized as M+ patients, and therefore AD was homogenously indicated in patients with N2 involvement irrespective of tumor dimension or infiltration. Another limitation was regarding the histological sub-classification for adenocarcinoma patients, which was not collected. Although we studied quite a homogeneous population of patients with single-station involvement, we believe it is the principal factor affecting the prognosis in these patients; it would be interesting to evaluate if different adenocarcinoma patterns also influence the prognosis in these patients. In addition, an appropriate analysis of patterns is also necessary to review pathological specimens for accurate adenocarcinoma classification; this could be the objective of specific studies.

## 5. Conclusions

Our study confirms that lymph node factors such as the number of involved nodes, skip metastases, and in particular, the lymph node ratio are good prognosticators in patients with single-station mediastinal involvement. Adjuvant therapy continues to have a fundamental role in the case of multiple lymph node involvement, while its role in single metastatic nodes needs to be clarified.

## Figures and Tables

**Table 1:** Clinical and pathological factors and lymph node characteristics.

CHARACTERISTIC	NUMBER
Age (years)	66.7 ± 15.2
Sex	
Male	131 (61%)
Female	82 (39%)
Tumor location	
Right upper lobe	81 (38.1%)
Right lower lobe	39 (18.4%)
Middle lobe	4 (1.9%)
Left upper lobe	66 (30.9%)
Left lower lobe	23 (10.7%)
Surgery	
Lobectomy	183 (85.4%)
Bilobectomy	12 (5.6%)
Pneumonectomy	18 (9%)
pT	
1	48 (22.5%)
2	126 (59.1%)
3–4	39 (18.4%)
Lymphadenectomy	
# Resected Nodes (median)	17 (range 3–50)
# Metastastic Nodes (median)	3 (range 1–21)
# Resected mediastinal node station (median)	3 (range 1–6)
Node ratio >10	143 (67.1%)
Node ratio <10	70 (32.9%)
Skip metastases	98 (46%)
Single metastatic node	63 (29.5%)
cN1	17 (7.9%)
cN2	39 (18.3%)
Adjuvant Therapy	
No	50 (23.4%)
Yes	149 (69.9%)
Missing data	14 (6.7%)

**Table 2:** Univariable and multivariable analyses. HR, hazard ratio; CI, confidence interval.

Variable	Disease-Free Survival	Overall Survival		
	Univariable	Univariable	Multivariable	
	*p* Value	*p* Value	*p* Value	HR (95% C.I.)
Sex	0.848	0.558	-	-
Age	0.124	0.009	0.003	1.802 (1.220–2.662)
Number N1 stations	0.707	0.003	ns	ns
Number of resected nodes	0.693	0.474	-	-
Number of metastatic nodes	0.817	0.009	ns	ns
Lymph node ratio	0.504	0.003	0.001	2.761 (1.683–4.530)
Skip metastases	0.192	0.005	ns	ns
pT stage	0.975	0.249	-	-
Tumor grading	0.024	0.401	-	-
Adjuvant therapy	0.521	0.143	-	-
Surgery (lobectomy vs. other)	0.172	0.077	-	-

## Data Availability

Data sharing is not applicable to this article because every institution is the owner of their data.

## Supplementary Materials

The following are available online at https://www.mdpi.com/2072-6694/13/6/1326/s1, Table S1: univariable and multivariable analysis in N2a1 patients, Table S2: univariable and multivariable analysis in N2a2 patients.
