# Supplementary material for: Survival Analysis in Single N2 Station Lung Adenocarcinoma: The Prognostic Role of Involved Lymph Nodes and Adjuvant Therapy
Source: Cancers (Basel). 2021 Mar 16;13(6):1326. doi: 10.3390/cancers13061326 (PMC7998125; doi:10.3390/cancers13061326)
Supplement: Supplementary file 1 [file cancers-13-01326-s001.pdf]

cancers

Article

Survival Analysis in Single N2 Station Lung Adenocarcinoma: The Prognostic Role of Involved Lymph Nodes and Adjuvant Therapy

Marco Chiappetta [1,2,*], Filippo Lococo [1,2,] ,Giovanni Leuzzi [3] , Isabella Sperduti [4], Emilio Bria [1,5], Leonardo Petracca Ciavarella [1,2], Felice Mucilli [6], PierLuigi Filosso [7], Giovannibattista Ratto [8], Lorenzo Spaggiari [9],Francesco Facciolo [10] and Stefano Margaritora [1,2]

[1.] , Università Cattolica del Sacro Cuore, 00168 Rome, Italy; marco.chiappetta@policlinicogemelli.it (M.C.); emilio.bria@policlinicogemelli.it (E.B.); leonardo.petracca@gmail.com (L.P.C.) stefano.margaritora@policlinicogemelli.it (S.M.)
[2] Thoracic Surgery, Fondazione Policlinico Universitario A. Gemelli IRCCS, 00168 Rome, Italy
[3] Thoracic Surgery Unit Fondazione IRCCS Istituto Nazionale dei Tumori, 20133 Milan, Italy; giovanni.leuzzi@istitutotumori.mi.it
[4] Biostatistics, Regina Elena National Cancer Institute - IRCCS, 00100 Rome, Italy; isabella.sperduti@ifo.gov.it
[5] Medical Oncology, IRCCS Fondazione Policlinico Universitario A. Gemelli, 00168 Rome, Italy
[6] Department of General and Thoracic Surgery, University Hospital "SS. Annunziata", 66100 Chieti, Italy; fmucilli@unich.it
[7] Department of Thoracic Surgery, University of Turin, San Giovanni Battista Hospital, 10126 Turin, Italy; pierluigi.filosso@unito.it
[8] Division of Thoracic Surgery, IRCCS AOU "San Martino" IST, 16132 Genoa, Italy; giovannibattista.ratto@gmail.com
[9] Thoracic Surgery Division, European Institute of Oncology, University of Milan, 20141 Milan, Italy; lorenzo.spaggiari@ieo.it
[10] Thoracic Surgery, Regina Elena National Cancer Institute, 00100 Rome, Italy; francesco.facciolo@ifo.gov.it
* Correspondence: marco.chiappetta@policlinicogemelli.it

Citation: Chiappetta, M.; Lococo, F.; Sperduti, I.; Bria, E.; Ciavarella, L.P.; Mucilli, F.; Filosso, P.; Ratto, G.; Spaggiari, L.; Margaritora, S.; et al. Survival Analysis in Single N2 Station Lung Adenocarcinoma: The Prognostic Role of Involved Lymph Nodes and Adjuvant Therapy. *Cancers* **2021**, *13*, 1326. https://doi.org/10.3390/cancers13061326

Academic Editor: Tetsuya Mitsudomi and Yasushi Shintani

Received: 12 February 2021
Accepted: 13 March 2021
Published: 16 March 2021

Publisher's Note: MDPI stays neutral with regard to jurisdictional claims in published maps and institutional affiliations.

Copyright: © 2021 by the authors. Licensee MDPI, Basel, Switzerland. This article is an open access article distributed under the terms and conditions of the Creative Commons Attribution (CC BY) license (http://creativecommons.org/licenses/by/4.0/).

Supplementary

Table S1. Univariable and multivariable analysis in N2a1 patients.

VARIABLE	DISEASE FREE SURVIVAL	OVERALL SURVIVAL		
	UNIVARIABLE	UNIVARIABLE	MULTIVARIABLE	
	p Value	*p* Value	*p* Value	HR (95% C.I.)
**Sex**	0.872	0.904	-	-
**Age**	0.613	0.413	-	-
**Number of resected nodes**	0.684	0.254	-	-
**Number of metastatic nodes**	0.437	0.290	-	-
**Lymph node ratio**	0.424	0.002	0.003	2.734 (1.417–5.277)
**pT stage**	0.400	0.039	0.050	2.136 (1.001–4.557)
**Tumor grading**	0.410	0.451	-	-
**Adjuvant therapy**	0.170	0.938	-	-
**Surgery (lobectomy vs other)**	0.009	0.936	-	-

Table S2. Univariable and multivariable analysis in N2a2 patients.

VARIABLE	DISEASE FREE SURVIVAL	OVERALL SURVIVAL		
	UNIVARIABLE	UNIVARIABLE	MULTIVARIABLE	
	p Value	*p* Value	*p* Value	HR (95% C.I.)
**Sex**	0.969	0.520	-	-
**Age**	0.10	0.002	0.009	2.013 (1.194–3.393)
**Number N1 stations**	0.161	0.403	-	-
**Number of resected nodes**	0.940	0.946	-	-
**Number of metastatic nodes**	0.351	0.463	-	-
**Lymph node ratio**	0.712	0.598	-	-
**pT stage**	0.563	0.898	-	-
**Tumor grading**	0.021	0.960	-	-
**Adjuvant therapy**	0.037	0.022	ns	ns
**Surgery (lobectomy vs other)**	0.816	0.065	-	-
